# Valorization of River Sediments in Sustainable Cementitious Gel Materials: A Review of Characteristics, Activation, and Performance

**DOI:** 10.3390/gels11090755

**Published:** 2025-09-18

**Authors:** Yuanxun Zheng, Yuxiao Xie, Yu Zhang, Cong Wan, Li Miao, Peng Zhang

**Affiliations:** 1 School of Water Conservancy and Transportation, Zhengzhou University, Zhengzhou 450001, China; yxzheng@zzu.edu.cn (Y.Z.); yuxiaoxie2023@163.com (Y.X.); wancong0518@163.com (C.W.); zhangpeng@zzu.edu.cn (P.Z.); 2 State Key Laboratory of Tunnel Boring Machine and Intelligent Operations, Zhengzhou 450001, China; 3 School of Civil Engineering, Harbin Institute of Technology, Harbin 150090, China

**Keywords:** river sediments, activation techniques, optimization of concrete properties, low-carbon building materials, proportion design methods

## Abstract

River sediments have attracted increasing attention as alternative raw materials for sustainable cementitious materials due to their abundant availability and silica–alumina-rich composition. In this study, a systematic literature search was conducted in Web of Science and Google Scholar using combinations of the keywords “river sediment,” “cementitious materials,” “activation,” and “pozzolanic activity,” covering publications up to July 2025. In addition, a citation network tool (Connected Papers) was employed to trace related works and ensure comprehensive coverage of emerging studies. This review systematically examines the properties of river sediments from diverse regions, along with activation and modification techniques such as alkali/acid activation, thermal calcination, and mechanical milling. Their applications in various cementitious systems are analyzed, with mix design models compared to elucidate the effects of replacing fine aggregates, coarse aggregates, and cement on workability, strength, and durability. Multi-scale characterization via XRD, FTIR, and TG-DSC reveals the mechanisms of C–S–H and C–A–S–H gel formation, pore refinement, and interfacial transition zone densification. The review highlights three key findings: (1) moderate sediment replacement (20–30%) improves strength without compromising flowability; (2) alkali–water glass activation and calcination at 600–850 °C effectively enhance pozzolanic activity; and (3) combining the minimum paste thickness theory with additives such as water reducers, fibers, or biochar enables high-performance and low-carbon concrete design. This review provides a comprehensive theoretical foundation and technical pathway for the high-value utilization of river sediments, carbon reduction in concrete, and sustainable resource recycling.

## 1. Introduction

### 1.1. Research Background

Amid growing global concerns over resource scarcity and environmental sustainability, the construction materials industry is undergoing a significant green transformation. As the most widely used human-made material, concrete is under scrutiny for its high consumption of natural resources and associated carbon emissions during production and use [1,2]. Over time, large-scale extraction of natural sand, gravel, and cement has led to resource depletion and ecological degradation. Moreover, cement production is energy-intensive and emission-heavy, making it a key target for carbon peaking and neutrality efforts [3,4]. Consequently, developing low-carbon, high-performance, and sustainable alternative raw materials has become a global research focus [5].

**Figure 1:**
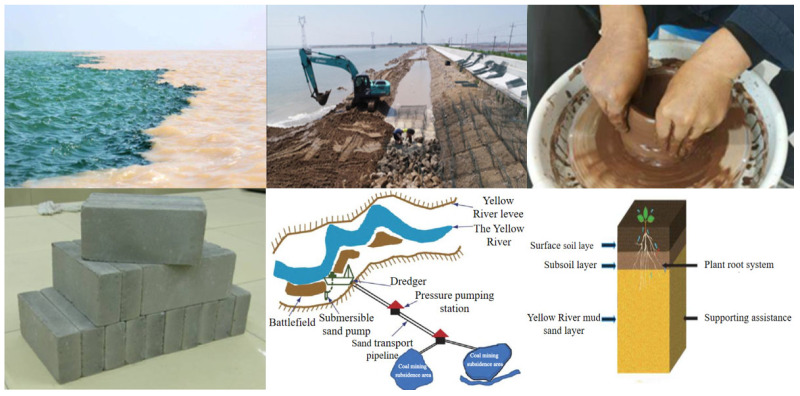
Yellow River sediment resource utilization.

River sediment, generated in large volumes through dredging during urban and natural river management, often exceeds hundreds of millions of tons annually. However, most of it remains unused, occupying land and posing environmental risks such as heavy metal leaching [6,7,8]. Compositionally, river sediments contain fine particles, clay minerals, and reactive silica–alumina components, showing similarities with traditional concrete materials in terms of gradation and mineralogy [9,10,11]. This has prompted interest in their use as fine aggregates, partial cement replacements, or auxiliary admixtures, offering a pathway for transforming waste into a resource and aligning environmental governance with material recycling goals [11,12]. The existing literature confirms that using sediments in ternary cementitious systems is a mature and promising research direction. Several studies have successfully applied sediments in ternary blends, showing improved mechanical properties and refined microstructures [13,14]. To date, various applications of river sediments have yielded promising results: modification into flood control materials [15]; use as pavement base and sub-base layers [16]; soil remediation and land reconstruction in low- and medium-yield sandy areas along the Yellow River, where optimized filling strategies have been developed [17,18,19]; and production of recycled bricks [20,21], lightweight aggregates [22], and ceramic granules [23] (Figure 1).

**Figure 2:**
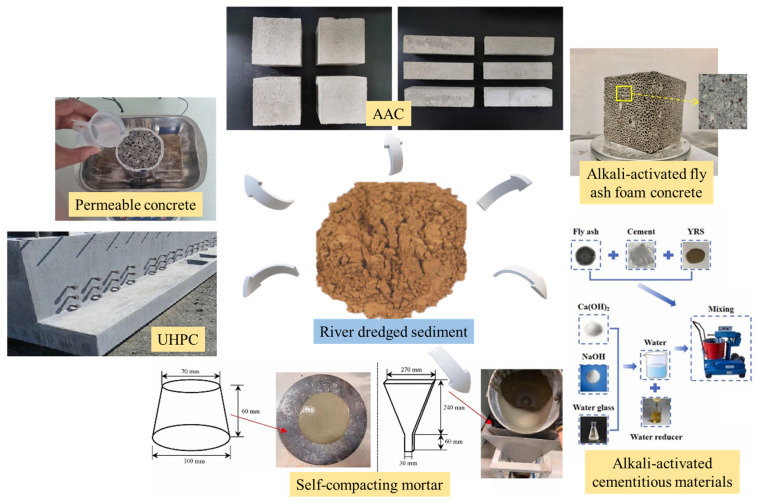
Application of river sediment in concrete (Permeable concrete [24], AAC [25], UHPC [26], Alkali-activated fly ash foam concrete [27]; Self-compacting mortar [28]; Alkali-activated cementitious materials [29]).

Based on sediment characteristics and engineering requirements, researchers have developed sediment-based concrete for a wide range of applications. These include pervious concrete, lightweight concrete, foam concrete, self-compacting mortar, polymer mortar, autoclaved aerated concrete (AAC), ultra-high-performance concrete (UHPC), and engineered cementitious composites (ECCs), as shown in Figure 2. In these applications, sediments function either as a replacement for sand or as a substitute for cementitious components.

Although river sediments have promising application potential, their use in concrete still faces multiple challenges due to variability in physicochemical properties, high impurity content, and low pozzolanic activity [26]. Sediments formed under different geographical and hydrological conditions exhibit significant differences in particle size distribution, mineral composition, and organic content, which directly influence their suitability for concrete applications [30,31]. Moreover, high moisture content, low reactivity, and the presence of humus or heavy metals can adversely affect workability, strength, and durability if not properly treated [32]. Therefore, identifying key physical properties, establishing applicability evaluation criteria, and enhancing reactivity through targeted techniques are essential to advancing sediment-based engineering applications. Recent studies have explored activation strategies to address these issues. Thermal treatment disrupts crystal structures and releases active phases [33,34]; alkali activation promotes reconstruction of the silica–alumina skeleton and formation of binding gels [35]; and mechanical milling increases surface area and exposes reactive sites [36,37]. These methods help overcome the limitations of weak cementitious activity.

However, current research in this field lacks a systematic and multi-scale framework, which limits the efficient, value-added utilization of river sediments in concrete. The main research gaps are (1) a reliance on isolated case studies rather than comparative analyses to establish general principles; (2) an insufficient understanding of microstructural evolution and interface interactions; (3) low substitution levels that hinder practical application; and (4) a shortage of life cycle assessments validating environmental and economic benefits. And, current research still faces several limitations: (1) most studies focus on single sediment types, lacking systematic comparisons to support universal mix design systems; (2) emphasis is placed on mechanical performance, while microstructural evolution and interfacial transition mechanisms remain insufficiently explored; (3) substitution levels are typically low and fail to meet performance thresholds required for real-world application; and (4) life cycle-based environmental and economic assessments are scarce, limiting large-scale adoption. In this context, systematically evaluating multi-path utilization strategies is essential. Such efforts can reveal the fundamental interactions between sediment particle characteristics, mineral composition, and cementitious systems, offering theoretical and technical support for the development of green building materials. A comprehensive analysis of sediment-based composites—from rheology and hydration to microstructure and durability—can enable performance optimization and functional material design. Furthermore, integrating life cycle and carbon footprint assessments will support the transformation of sediments from low-value alternatives to high-performance green resources. This review focuses on the use of river sediments in concrete, summarizing their properties, activation and modification methods, substitution strategies, and performance enhancement mechanisms. It also examines their role in hydration, microstructure evolution, and durability, while proposing future research directions aligned with engineering demands and low-carbon goals. The findings aim to provide a systematic theoretical basis and technical framework for the high-value utilization of river sediments and contribute to the sustainable transformation of the concrete industry.

### 1.2. Methodology

This systematic review was conducted using a structured and reproducible protocol to identify and analyze relevant studies on the use of river sediments in cementitious materials, with a focus on activation mechanisms and pozzolanic activity. The search was carried out primarily using the Web of Science Core Collection and Google Scholar. To ensure comprehensive coverage, an extensive set of keywords and Boolean combinations was employed. The search strings included the following: (“river sediment” OR “dredged sediment” OR “silt”) AND (“cementitious material” OR “alkali-activated” OR “geopolymer” OR “supplementary cementitious material”) AND (“activator” OR “mechanochemical” OR “thermal treatment”) AND (“volcanic” OR “pozzolan” OR “ash”). The search covered all publications in these databases up to July 2025.

The study selection followed predefined criteria. Publications were included if they (1) focused on river/dredged sediments as raw materials; (2) examined their use in cementitious or alkali-activated binders; (3) detailed activation methods (mechanical, thermal, or chemical); and (4) discussed pozzolanic/volcanic activity or reaction mechanisms. Exclusion criteria included conference abstracts, editorials, and book chapters without original experimental data. Screening was conducted in two phases. First, all retrieved articles were screened by title and abstract. Then, the full texts of potentially relevant articles were assessed for eligibility. To capture emerging studies not yet indexed, the Connected Papers tool was used to trace backward and forward citations of key articles, ensuring comprehensive coverage. A standardized data extraction form was used to collect information from the included studies. The initial search retrieved 369 records. After title and abstract screening, 188 documents proceeded to full-text evaluation. Ultimately, 149 studies met the criteria and were included for data extraction and analysis. Key data included (1) sediment characteristics (e.g., origin and chemical/mineralogical composition); (2) activation process details (e.g., method and parameters); (3) composite mix designs (e.g., binder composition and replacement ratios); (4) curing conditions; and (5) measured properties (e.g., strength, reaction products, and microstructure). Given methodological heterogeneity, a narrative synthesis was adopted. Findings were thematically organized to compare the effects of different activation techniques on sediment pozzolanic activity and to discuss reaction mechanisms.

While every effort was made to ensure comprehensiveness, this review has limitations. Differences in activation and testing protocols restricted the possibility of quantitative meta-analysis. Despite multiple search strategies, some recent or unpublished work may not have been captured.

## 2. Physical Characteristics and Activation Measures of River Sediments

### 2.1. Fundamental Characteristics

#### 2.1.1. Physical Characteristics

Before dredging and utilizing river sediments, the sediments can be characterized based on the contaminant content, organic matter content and particle size distribution of the deposits, and statistical analysis of the resulting database can help to categorize these sediments, which can then be used to experimentally quantify the impact of the various sediments on concrete in a more efficient manner [38]. In some areas, the newly dredged sediments inevitably contain pollutants such as waste metals, and the total pollutant content can be detected by Inductive Coupled Plasma [39].

**Figure 3:**
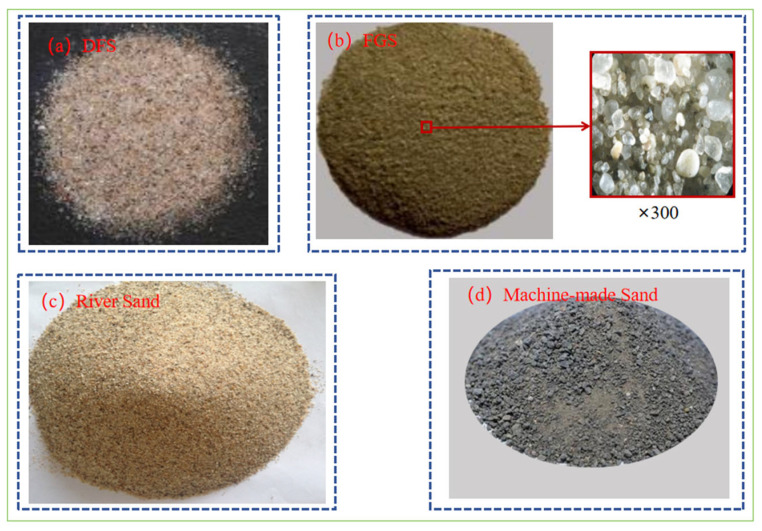
Comparison of material morphology of different sands. (**a**) Deposited fine sand (DFS) [40], (**b**) fine-grained sediments (FGS) [41], and its 300× micrograph [29]; (**c**) river sand; and (**d**) machine-made sand.

**Figure 4:**
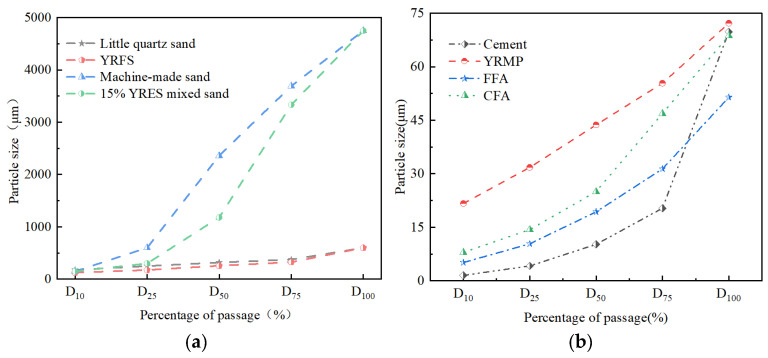
Particle size distribution of different types of sand/particles. (**a**) Particle size distribution of small quartz sand, deposited fine sand, mechanism sand, and a blend of 15% dosed Yellow River Fine Sand(YRFS) and mechanism sand; (**b**) particle size distribution of ordinary silicate cement [42], low calcium fly ash (FFA) [43], high calcium fly ash [44] (CFA), and Yellow River Micro-Powder (YRMP) [40].

The sediments were classified into deposited fine sand and fine-grained sediments according to the particle size, and the appearance comparison with ordinary river sand as well as mechanism sand as shown in Figure 3, which has a big difference in morphology and color. In terms of particle size distribution, the particle size of fine sand is similar to that of small quartz sand, and the particle size of micro-powder is slightly larger than that of cement and other auxiliary cementitious materials, as shown in Figure 4a,b. Additionally, a few studies have examined larger sediment particles used as coarse aggregates in cementitious materials [24,39]. Since aggregate properties—such as moisture content, water absorption, and density—strongly affect cementitious performance, Table 1 summarizes their physical properties. Sedimentary micropowders are widely used as cement substitutes, and analyzing their chemical and mineralogical composition is essential for understanding macro- and micro-scale behavior. This topic is discussed in the next section. According to Table 1, sediment moisture content ranges from 0.065% to 10.69%, which directly influences the effective water-cement ratio. Water absorption also strongly affects slump and other physical properties. Highly absorbent aggregates often have high porosity and low strength, creating weaknesses in concrete. When absorption exceeds 2.5%, rapid uptake of mixing water leads to slump loss. In addition, highly absorbent materials usually have low density, which reduces hardened concrete strength and durability. Therefore, when such sediments are used, pretreatment or water-cement ratio adjustment is required.

#### 2.1.2. Chemical Composition

**Figure 5:**
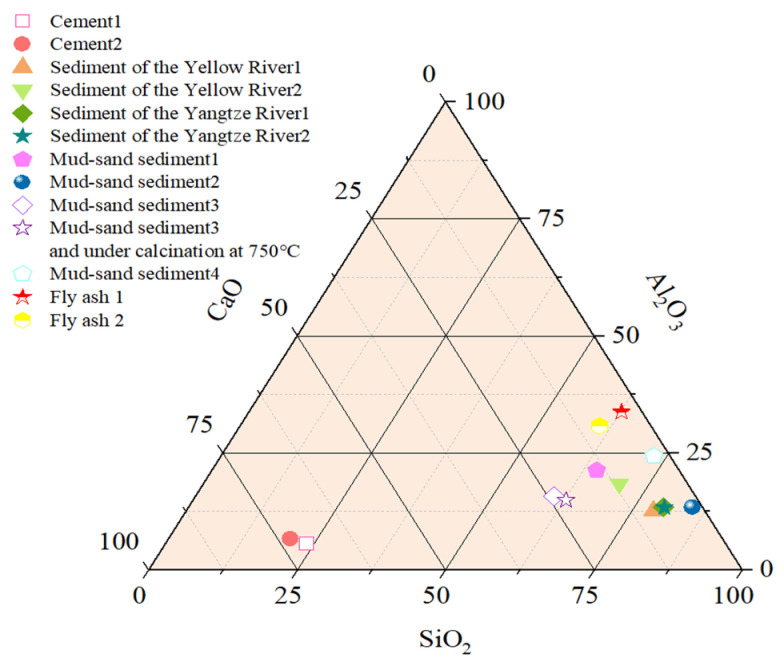
Comparison of chemical composition of different materials [5,18,23,25,29,45,46,47,48].

X-ray fluorescence analysis (Table 2) shows that the chemical composition of river sediments differs significantly from that of cement. Unlike cement, which is CaO-rich, sediments contain higher SiO_2_ and moderate Al_2_O_3_, resembling fly ash in composition [18,46]. As illustrated in Figure 5, sediments exhibit intermediate characteristics: their SiO_2_ and Al_2_O_3_ contents are higher than those of cement but lower than those of fly ash, while CaO content is lower than cement but higher than fly ash. Comparison of aggregate composition in Table 2 with ASTM C618 shows that the total SiO_2_, Al_2_O_3_, and Fe_2_O_3_ in most sediments exceeds 70%, meeting the minimum requirement for type N pozzolan in ASTM C618. This “medium silica–alumina with moderate calcium” profile indicates potential pozzolanic activity and Ca-supplement capacity, making sediments suitable as supplementary cementitious materials, especially for partial cement replacement or use with activators in low-carbon concrete.

Given these characteristics, deposited fine sand can feasibly replace or blend with river sand, manufactured sand, or quartz sand as a fine aggregate. This is particularly advantageous, as manufactured and quartz sands often suffer from high porosity, poor gradation, low workability, and high angularity [49,50]. Sedimentary sand, being finer and comparable to quartz sand, is environmentally favorable for partial substitution in concrete [51,52,53,54]. Currently, most studies treat deposited fine sand as inert aggregate, optimizing mix designs with limited dosages. However, due to its low reactivity, performance improvement remains restricted [55,56], and low replacement levels reduce economic feasibility [57,58]. In contrast, finer sedimentary powders show greater reuse potential. As shown in Table 2, sediments are primarily composed of SiO_2_ and Al_2_O_3_, with lower levels of CaO, Fe_2_O_3_, K_2_O, MgO, and Na_2_O. As aluminosilicate-rich materials, they possess theoretical potential for geopolymerization under suitable conditions. Activating this reactivity could enable cement substitution and offer a new pathway for sediment resource utilization.

#### 2.1.3. Mineral Composition

Given the growing focus on ternary cement and the synergistic effects of calcined clay and other cementitious materials, it is important to discuss the mineralogical composition of sediments to demonstrate their potential in producing alternative cement.

Subramanian et al. [59] investigated sediments from the Yamuna River, a Himalayan tributary of the Ganges, and found that quartz and feldspar were the most abundant minerals. The clay mineral assemblage was mainly illite and chlorite, with minor kaolinite and montmorillonite. Hamdy et al. [60] studied the entire Ganges system and reported that the clay fraction was dominated by illite, followed by smectite and kaolinite/chlorite. Li et al. [61] studied rivers in Taiwan, China, and found that sediments mainly consisted of illite, smectite (montmorillonite-group), chlorite, and kaolinite, with smectite reaching 40–80% in some rivers. Singh et al. [62] studied the Kaveri River in southern India and found the main clay minerals to be montmorillonite, kaolinite, and illite.

These studies show that quartz and feldspar are the dominant light minerals in sand and silt fractions, typical products of continental crust weathering. Analysis of sediment composition shows high silica and alumina, indicating quartz (SiO_2_), clay minerals such as illite and kaolinite, and possibly feldspathic phases [63,64]. These aluminosilicates are critical for pozzolanic behavior, as they react with Ca(OH)_2_ in cementitious systems to form C–S–H phases [65]. The moderate calcium content supports self-cementing potential under activation, bridging traditional pozzolans and latent hydraulic materials such as GGBS [66]. The fine particle size distribution enhances reactivity by providing nucleation sites for hydration and promoting microstructural densification [67]. Low contents of K_2_O, Na_2_O, and MgO reduce risks of expansion and ASR cracking, supporting safe use as a construction material [68].

In summary, river sediments rich in reactive silica and alumina, with supplementary calcium, show pozzolanic potential and alkali activation compatibility, supporting their use as sustainable SCMs in modern concrete.

### 2.2. Activation Measures for Fine-Grained River Sediments

Li et al. [69] demonstrated that Pisha sandstone—the dominant mineral resource within Yellow River sediments—constitutes a distinctive clay-rich formation. This lithology comprises non-crystalline clay minerals (montmorillonite, illite, mica, etc.) and crystalline phases (feldspar, quartz, calcite, etc.), thereby providing evidence for the low pozzolanic activity inherent to fine-grained river sediments. To enable its use as a cement substitute, activation is necessary to enhance its reactivity. Properly activated sediment powder—via calcination or mechanical treatment—transforms kaolinite and other clays into amorphous metakaolin, which, in the presence of Ca(OH)_2_ and water, forms C–S–H and C–A–H gels. This process effectively regenerates micro-scale cementitious phases and improves matrix strength and densification [70].

#### 2.2.1. Chemical Excitation Activation

(1) Categories of chemical excitants

Alkali activators enhance reactivity by disrupting mineral structures and releasing active components [29]. Among these, NaOH and water glass are the most common. Li et al. [69] demonstrated that using NaOH and water glass (modulus 3) significantly increased the compressive strength of Pisha sandstone. Water glass alone can also activate sediments [71]. Jiang et al. [27] and Jing et al. [72] found that mono-doping with 10–15% Ca(OH)_2_ optimized pozzolanic reactivity. Ze et al. [29] conducted a study on the preparation of Yellow River sediment/fly ash/cement-based alkali-activated cementitious materials for coal mine filling. The study compared different activators, including Ca(OH)_2_, NaOH, sodium silicate (water glass), and their combinations. All these activators promoted more complete reactions of cement, fly ash, and Yellow River sediment, which led to the formation of additional C–S–H and C–A–S–H gels. Among them, the combination of NaOH and sodium silicate proved most effective in refining pores and improving the microstructure. However, Junakova et al. [73] argued that NaOH alone is ineffective for activating sediments. Regarding this contradictory phenomenon, research by Palomo et al. [74] revealed that excessive NaOH use, due to its high alkalinity, increases the alkalinity of the hydration solution and reduces the hydration of anhydrous silicates, thereby decreasing compressive strength.

Salt activators also show promise. Hai et al. [57] used a 6% mixture of CaCl_2_ and water glass (1:1) to activate sediments. Ca^2+^ exchanged with ions in clay minerals, releasing soluble Si and Al, while reacting with C_3_A to form hydrated calcium chloroaluminate, thus enhancing strength. CaCl_2_ also accelerates feldspar hydrolysis, while water glass fills pores with silica and calcium silicate gels. However, the chloride ions in CaCl_2_ may induce long-term durability issues. Liu et al. [21] proposed sodium bisulfite as an activator, though its mechanism requires further clarification.

In addition, acidic activation has gained attention. Wang [75] studied Yellow River sediment and used acidic activators to replace cement in cement–sand specimens of the same particle size. The results showed that acid activation provided stable strength, improved energy efficiency, and offered environmental benefits while avoiding durability problems related to alkali. Phosphoric acid, acetic acid, and hydrochloric acid corroded particle surfaces, increased surface area, and promoted gel formation. Acid–base synergistic activation further accelerated ion release and hydration, enhanced density, inhibited salt migration, and effectively mitigated alkali–silica reaction.

(2) Activation reaction mechanisms

**Figure 6:**
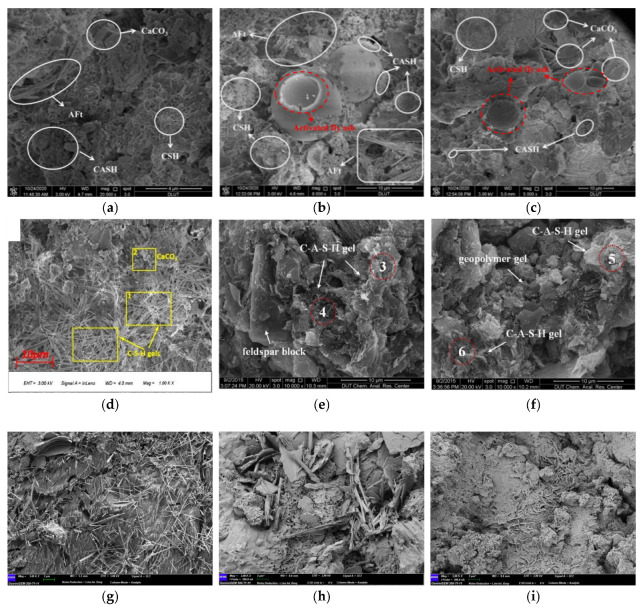
Microscopic morphology of cementitious materials after adding different chemical excitants. (**a**) NaOH [29]; (**b**) water glass [29]; (**c**) water glass + NaOH [29]; (**d**) Ca(OH)_2_ [27]; (**e**) Na_2_CO_3_ [71]; (**f**) Na_2_SO_4_ [71]; (**g**) 1.5% sodium pyrophosphate [75]; (**h**) 1% acetic acid [75]; and (**i**) 1.5% hydrochloric acid [75]. (The scale bar length in (**g**,**h**) is 2 μm.)

As shown in Figure 6a–c [29], under alkaline excitation, the matrix incorporating activators exhibited a denser structure than the blank matrix, with fly ash and YRS particles encapsulated by C–S–H and C–A–S–H gels. Similarly, in Figure 6d [27], the addition of Ca(OH)_2_ led to the formation of abundant honeycomb and fibrous C–S–H gels, enhancing matrix densification and reducing average pore size. In the Na_2_CO_3_ and Na_2_SO_4_ systems (Figure 6e,f), hydration between sediment and activator was limited. The resulting structure was loose, with flocculated geopolymer gels coexisting with unreacted matrix and feldspar clasts wrapped in gels. In the acidic systems (Figure 6g–i [75]), the 1.5% phosphoric acid group produced abundant needle-like and flocculent hydration products, forming a dense and continuous gel skeleton. These products included C–S–H gels, Ca(OH)_2_ crystals, and Ca_3_(PO_4_)_2_, effectively filling pores and increasing strength. In contrast, the 1% acetic acid and 1.5% hydrochloric acid groups also generated C–S–H gels and calcite, but with lower product content and poor connectivity, resulting in weaker excitation effects than the phosphoric acid group.

**Figure 7:**
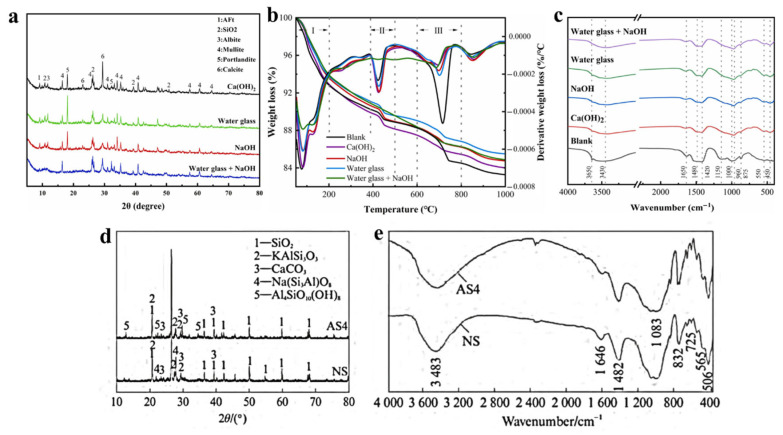
XRD, TG and FTIR patterns of the prepared filling materials from Yellow River sediments under different excitants. (**a**–**c**) Ca(OH)_2_, NaOH, water glass, water glass + NaOH [29]; (**d**,**e**) CaCl_2_ + water glass [75].

The hydration products and microstructures of the fillers changed significantly under different activators. XRD results showed that doping with Ca(OH)_2_, NaOH, water glass, or CaCl_2_ increased C–S–H and C–A–S–H gel formation. Co-doping with NaOH and water glass depleted Ca(OH)_2_, while excitation with CaCl_2_ eliminated natrium feldspar peaks and introduced kaolinite peaks, indicating ion exchange and mineral transformation (Figure 7a,d). TG analysis confirmed that activators enhanced hydration reactions. Dehydration of products increased with Ca(OH)_2_ depletion, CaCO_3_ content decreased, and gel production rose significantly (Figure 7b). FTIR results showed that doping eliminated Ca(OH)_2_ characteristic peaks, caused shifts in Si–O peaks, and CaCl_2_ enhanced O–H absorption peaks, indicating increased hydroxyl content that promoted particle bonding. Overall, excitants significantly improved hydration and microstructural densification (Figure 7c,e) [29,75].

The above results indicate significant differences in the efficacy of chemical activators. The choice of the optimal activator depends on its overall performance in the chain of “destroying the original structure, providing reactive components, and forming stable products.” NaOH–water glass enhances gel formation and structure through synergistic effects in alkaline environments. Phosphoric acid, in contrast, produces a strong and durable microstructure through corrosion and stable precipitation in acidic conditions. However, the Cl^−^ introduced by CaCl_2_ induces steel corrosion, posing a serious threat to long-term durability and greatly limiting its practical application.

In summary, the microstructure and activity of river sediments were improved to a great extent under the action of chemical exciters, in which the activation principles of acidic and alkaline exciters differed. Currently, there is a lack of systematic research on the selection of river sediments activating agents, optimal modulus, optimal dosage, and their relationship with hydration characteristics and macro-microstructure, and the future should focus on exploring these aspects.

#### 2.2.2. Thermal Activation

**Figure 8:**
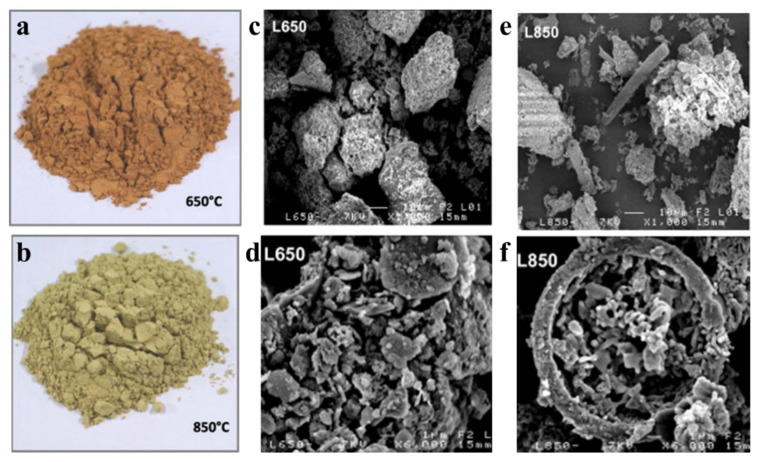
(**a**,**b**) **Visual aspect of sediment heated at 650 °C and 850 °C**; (**c**,**d**) SEM images at 650 °C with magnifications of 1kx and 6kx; (**e**,**f**) SEM images at 850 °C with magnifications of 1kx and 6kx [80].

Thermal activation enhances the pozzolanic activity of sediments by converting crystalline clay phases into an amorphous state [48,76]. For instance, Ferone et al. [46] calcined clay sediments from two reservoirs in southern Italy at 400 °C and 750 °C for 2 h at 10 °C/min. Li et al. [77] studied Pisha sandstone decomposition at 600 °C and 800 °C. Wang et al. [23] investigated the effect of calcination temperature on Yellow River sediment-based ceramic grains. To reduce energy use, Snellings et al. and Duc Chinh Chu et al. [78,79] applied flash calcination to treat sediments. Post-treatment, visible color differences between 650 °C and 850 °C (Figure 8a,b) suggested compositional changes. SEM images (Figure 8c–f) showed granular structures with surface recrystallization, indicating localized melting. Samples treated at 650 °C contained fine particles, while those at 850 °C showed melted and recrystallized fines [80].

**Figure 9:**
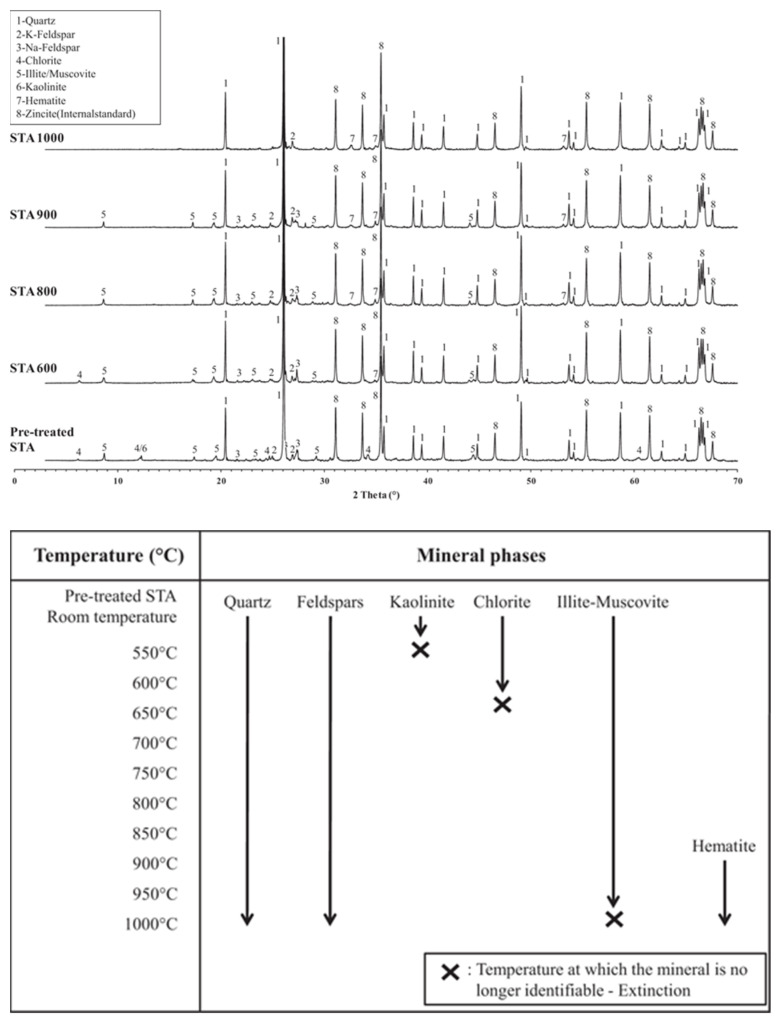
XRD patterns and identification of crystalline phases at different calcination temperatures [87].

Figure 9 presents XRD patterns at various calcination temperatures. Quartz remained thermally stable between 550 and 1000 °C. Feldspar showed peak shifts but incomplete decomposition at 1000 °C. Clay minerals, especially kaolinite, underwent major changes—its (001) peak disappeared above 550 °C, confirming dehydroxylation and the formation of amorphous metakaolinite (Al_2_O_3_·2SiO_2_), which improved pozzolanic activity [81,82]. Although reactivity increased up to 800 °C, temperatures above 950 °C reduced activity due to mullite crystallization. Chlorite began dehydroxylation at 650 °C and completed by 800 °C, indicating a narrow activation window [83]. Cook [84]. noted its recrystallization near 850 °C. Illite and muscovite were more thermally stable, with peaks present even at 950 °C, where they became most reactive [85,86]. Hematite was the only recrystallized mineral detected, with peaks appearing from 600 °C and intensifying at higher temperatures, indicating progressive crystallization. Thus, thermal treatment at 550–950 °C effectively activates clay-rich dredged sludge, with careful temperature control key to optimizing amorphous phase formation [46].

**Figure 10:**
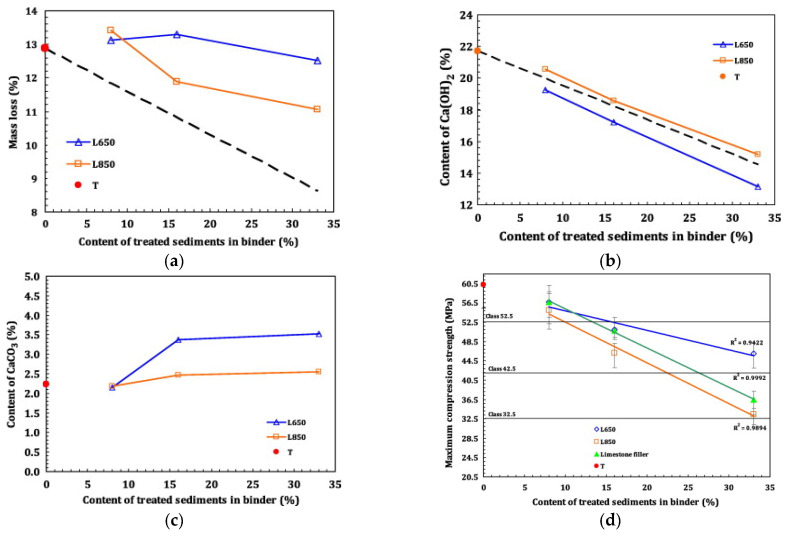
(**a**) Amount of bound water (between 105 °C and 400 °C) versus the amount of treated sediment in the slurry (The dashed line presents the amount of bound water corresponding to the part of Portland cement in the blended paste); (**b**) Amount of Ca(OH)_2_ in the slurry (by mass) versus the substitution ratio (The dashed line presents the amount of calcium hydroxide corresponding to the part of Portland cement in the blended paste); (**c**) Amount of CaCO_3_ in the slurry (by mass) versus the substitution ratio; and (**d**) 28-day compressive strength of the slurry versus the treated sediment and limestone filler substitution rates [80].

Figure 10a shows greater mass loss between 105 and 400 °C in mixtures with treated sediments than in Portland cement, indicating sediment involvement in hydration. In Figure 10b, 650 °C-treated samples fell below the dashed line, showing pozzolanic behavior [88]; 850 °C-treated samples were above the line due to calcite decarbonization forming reactive CaO. Figure 10c confirms lower CaCO_3_ in 850 °C samples, supporting Ca(OH)_2_ formation [80]. Figure 10d shows that at high substitution rates, 650 °C-treated samples exhibited higher compressive strength than limestone.

In summary, thermal activation alters sediment mineral structures, greatly enhancing activity without introducing external substances. For clay-rich minerals, the optimal calcination temperature is 550–950 °C, where amorphous transformation is highest and reactivity is strongest. This enhances early strength and long-term durability of the cementitious system. Samples treated at 850 °C produced highly active CaO, which promoted secondary hydration and generated more C–S–H gel, significantly improving matrix density and strength. Samples treated at 650 °C showed higher compressive resistance than limestone. Optimizing the calcination process achieves a balance between strength and durability, providing key technical support for high-performance cementitious materials. However, it requires high energy, large equipment, and may burn off active components. Chemical activation operates at room temperature, is simpler and adjustable, but may introduce impurities and environmental risks. Each method has advantages and limitations and should be selected based on application needs. A combined approach may also be considered [46].

#### 2.2.3. Mechanical Milling

Mechanical milling, as a physical activation method, effectively improves the sediment’s cementitious activity for partial cement replacement. Grinding increases specific surface area and exposes reactive sites, accelerating interactions with hydration products [89,90]. Kou et al. [5] and Beddaa et al. [24,39] found that grinding sediments can enhance hydration reactions by providing nucleation sites and promoting micro-flocculation. This improves packing density, pore structure, and durability. Junakova and Junak [73] further compared pure dry milling, co-milling with NaOH, with fly ash, and with both. NaOH alone had a limited effect, while composite treatments, especially those with fly ash, significantly enhanced sediment reactivity.

In summary, chemical activation operates at room temperature, is simple and adjustable, but may introduce impurities and environmental risks. Thermal activation fundamentally alters the crystalline structure, creating a stable amorphous phase with consistent reactivity, ideal for high-performance applications. Mechanical milling with additives such as biochar and fibers forms a “micro-cement + filler” composite, improving 28-day compressive strength by 10–20%. Mechanical milling enhances sediment reactivity and improves structure and performance through refinement and surface activation, providing strong support for sustainable construction. Each method has advantages and limitations, and selection should depend on application needs. A combined approach may also be considered.

#### 2.2.4. Pozzolanic Reactivity Assessment Methods

Activated dredged sediments are expected to exhibit pozzolanic activity and must be evaluated to confirm activation. Four evaluation methods are outlined below:

(1) A rapid evaluation method for the activity of pozzolanic materials was used, and the activity rate (*K_α_*) of pozzolanic materials was defined as the ratio of the total reactive SiO_2_ and Al_2_O_3_ (reacted with saturated limewater) to the total amount of SiO_2_ and Al_2_O_3_, as shown in Equation (1) [91].

(1) $$K_{\alpha }=\frac{{\mathrm{Active}(\mathrm{SiO}}_{2}{+\mathrm{Al}}_{2}O_{3})}{{\mathrm{Total}(\mathrm{SiO}}_{2}{+\mathrm{Al}}_{2}O_{3})}$$

(2) The Fratini test assesses pozzolanic reactivity by monitoring changes in OH^−^ and Ca^2+^ concentrations in a system comprising 20% mineral admixture and 80% CEM I cement. After sealing at 40 °C for 8–15 days, ion concentrations are compared with the Ca^2+^ solubility curve to determine activity [92].
(3) The strength activity index method evaluates mechanical performance by comparing the compressive strength of cement paste containing supplementary cementitious material with that of control paste made from ordinary Portland cement [87].
(4) Calorimetry is used to assess the effect of activated sedimentary pozzolanic material on cement hydration rate, thereby indirectly evaluating pozzolanic activity [87].

## 3. Performance-Enhancement Design for Sediment-Based Concrete

### 3.1. Mix Proportioning Methodology

River sediments typically have high water absorption and broad particle size distribution, making traditional mix design methods unsuitable [93]. Current approaches mainly rely on empirical or trial-and-error methods [20,94], which are inefficient and lack precision. Therefore, developing a scientific and efficient mix design method is essential for effective sediment utilization. This section reviews several mix design theories used for fine sand and ultrafine powder concrete and explores their applicability to river sediment concrete. The main methods are listed in Table 3:

Current mix design models fall into two categories: aggregate-based and slurry-based. Aggregate-based models optimize particle packing but often fail to address the high paste demand caused by fine sediments’ large surface area and high water absorption, limiting effectiveness. For example, Maherzi et al. [101] and Ennahal et al. [103] applied the PDM method to combine particle grades and minimize porosity. Jun et al. [26] optimized solid sizes with an improved Andreasen and Andersen model to design UHPC containing Yellow River ultrafine sand. However, these methods mainly focus on aggregate structure while neglecting paste dosage and distribution. This makes it difficult to resolve poor workability and low durability caused by high fines, large surface area, and high-water demand in river sediments. Slurry-based methods involve determining powder proportions, calculating aggregate voids, setting the average paste thickness (APT), and deriving component dosages. Studies show that adjusting parameters such as fine-to-coarse ratio, sand rate, water-cement ratio, fly ash/slag content, and water-reducer dosage reduces paste content, lowering cost and carbon emissions [104,105]. For instance, Luan et al. [105] applied this theory to design dredged sand concrete mixes by adjusting target strength, APT, and sand content. They found that increasing APT first enhances compressive strength before it declines, while workability keeps improving. This demonstrates the feasibility and effectiveness of the minimum paste theory for dredged ultrafine sand concrete. In contrast, paste-based methods directly regulate paste dosage and distribution. They offer tailored solutions for improving workability and durability in high-fines, high-absorption sediment concretes by minimizing excess paste while maintaining performance.

### 3.2. Performance Enhancement in Sediment-Functionalized Concrete

Although the fine-grained sediments are used as a high silica–alumina material, their activity is low and cannot directly participate in the hydration reaction. Although a series of activation measures have been taken, with the gradual increase in the dosage of its replacement cementitious material, the generation efficiency of hydration products such as C–S–H and C–A–S–H will still be affected, resulting in a gradual decrease in the macro-mechanical properties of the specimen. Deposited fine sand also introduces excessive defects, leading to the loss of properties when the substitution rate is too high [106]. Therefore, there is an urgent need to continue to investigate the performance enhancement mechanism of sediment-deposited concrete on this basis.

#### 3.2.1. Sediment as Partial Aggregate Replacement

The incorporation of river sediments as aggregates in concrete compensates for the lack of fines in washed mechanical sand, improves particle gradation, and enhances mechanical properties [45,107,108]. After particle size sorting, sediment can partially or fully replace natural coarse and fine aggregates [38]. Beddaa et al. [39] achieved C30 concrete by fully replacing aggregates with sediment, requiring only 5% more cement to meet the target strength; the dynamic modulus was comparable to the control. Junakova et al. [109] reported that substituting 20 wt% coarse sediment yields similar compressive strength to conventional concrete.

Sediment-based aggregates are now used in various cement systems to study the effects of different replacement rates. In alkali-activated systems, a 25% substitution of Yangtze dredged sand showed the best performance, improving compressive strength by about 5%, maintaining workability, and producing a denser interfacial zone [45,106]. Similarly, concrete designed with ultrafine dredged sand using the minimum slurry theory achieved the lowest porosity and highest strength at 25% substitution, confirming the feasibility of ultrafine sediment incorporation [105]. Sediment is also used as a polymer mortar aggregate. Maherzi et al. [101] optimized particle packing to reduce porosity, while Ennahal et al. [103] showed that polyester encapsulation stabilizes sediment ions and improves durability. Lightweight aggregates made from roasted sediments have low density and moderate water absorption, making them suitable for lightweight and self-compacting concrete with technical, ecological, and social benefits [22,26,110].

In summary, with proper particle size control and the use of admixtures or polymers, river sediments can replace fine, coarse, or full aggregates to improve performance and durability. The optimal replacement rate is typically 20–30%. For higher rates, adjustments such as modifying the water-cement ratio, adding superplasticizers, or using sintered lightweight aggregates are necessary, providing a practical approach for sediment resource utilization.

#### 3.2.2. Sediment as Partial Cementitious Material Replacement

(1) Chemical activation

As discussed in Section 2.2, various activation methods have been explored to induce pozzolanic activity in river sediments. Incorporating pozzolanic materials prolongs setting time, enhances Al and Si reactions to form (N,C)–A–S–H gels, improves hydration and mechanical properties, lowers cost, and reduces carbon emissions [77,111,112]. Activation is thus an effective way to enhance sediment concrete performance and will not be elaborated here.

(2) Performance-enhancing additives

To enhance the performance of dredged sediments as partial cement replacements, composite modification via external additives has gained attention. Functional additives can regulate hydration, microstructure, and mechanical properties across scales, improving applicability and durability.

Zhang et al. [113] showed that adding 0–2% biochar to sediment-based lightweight concrete promoted hydration and carbonation, improving microstructure and increasing 28-day compressive strength from 3.92 to 4.61 MPa. This effect was due to biochar’s porous structure and carbon content, which enhance nucleation and carbonation. Lang’s team explored the synergistic reinforcement of sediments with fibers and nanomaterials. Polypropylene, rice straw, and wheat straw fibers improved crack resistance and ductility via bridging effects [114,115]. Specifically, 3 mm polypropylene fibers enhanced interfacial bonding and provided strain-hardening behavior [114], while straw fiber doses above 0.5% reduced performance due to poor dispersion [115]. Nanomaterials such as nano-SiO_2_ and nano-MgO refined the pore structure and accelerated hydration, boosting strength by up to 40% [116]. Nano-MgO also improved volume stability due to its slight expansion [107]. Lang’s team proposed a fiber-nanomaterial synergy strategy: combining rice straw fiber and nano-SiO_2_ increased compressive and tensile strengths by 35% and 28%, respectively—better than using either alone [116]. Polypropylene fibers with nano-MgO further achieved both high strength and ductility [117]. This synergy arises from nanomaterials optimizing the matrix, fibers enhancing macro-properties, and improved interfacial transition zones.

In addition, moderate use of superplasticizers in blended cement systems slightly delays peak hydration but improves particle packing and workability. Especially at high sediment replacement rates (30–40%), superplasticizers help ensure compatibility and control air content [78].

In summary, external modification using functional additives significantly enhances the reactivity, strength, and durability of sediment-based cementitious materials through multiscale mechanisms, offering a critical path for their high-value application.

## 4. Comprehensive Performance Analysis of River-Sediment Concrete

### 4.1. Hydration Heat Release

**Figure 11:**
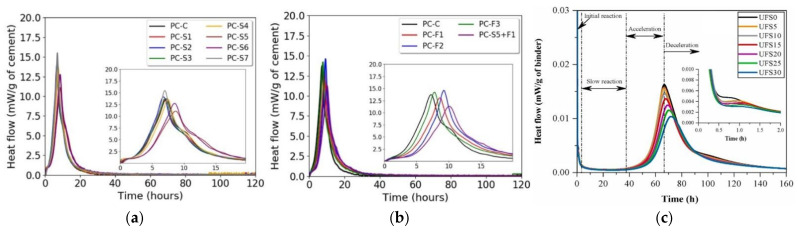
Hydration kinetics of concretes based on sediments: (**a**) sand sediment [24]; (**b**,**c**) fine sediment [24,28] (S1–S7: dredged sands from different areas of the same watershed; F1–F3: different dredged material powders pulverized at 105 °C and sieved through an 80 μm sieve; UFS: ultra-fine sediment).

Deposited fine sand does not slow down hydration and slightly increases total heat release, as shown in Figure 11a [24]. In contrast, fine-grained sediments significantly slow down hydration and reduce heat release due to their high soluble humus content. Humus is complex organic matter formed by the decomposition of animal and plant remains in river ecosystems. Beddaa et al. [24] reported that organic compounds in fine sediments—especially humic and fulvic acids—adsorb onto cement surfaces and hydration nucleation sites. This process delays the dissolution of cement clinker minerals and the growth of C–S–H gel. It slows hydration and reduces cumulative heat release. Its role is primarily physical filling rather than chemical participation, see Figure 11b [24]. Similarly, Hamideh et al. [28] found that increasing amounts of fine-grained sediments delayed the main exothermic peak, confirming its hydration-delaying effect, see Figure 11c. However, previous thermal analyses did not explain the change in intensity of the small shoulder peak after the main exothermic peak, which is generally attributed to continued C_3_A hydration. The influence of sediments on C_3_A hydration thus warrants further investigation.

### 4.2. Rheological Behavior

**Figure 12:**
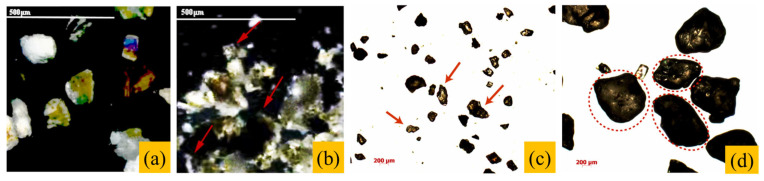
(**a**) Morphology of ultrafine dredged sand 1 and (**b**) mechanism sand [105]; (**c**) ultrafine dredged sand 2 and (**d**) river sand [45].

As shown in Figure 12a,c, ultrafine dredged sand particles are smaller and more rounded than the angular and irregularly shaped mechanism sand (Figure 12b) or the larger river sand (Figure 12d), both of which increase interparticle friction and reduce concrete fluidity [118]. According to the minimum paste thickness theory, even with increased dredged sand content, maintaining average paste thickness ensures improved slump, demonstrating the theory’s effectiveness. However, without controlling paste thickness, increasing the dredged sand lowers the slump [119].

**Figure 13:**
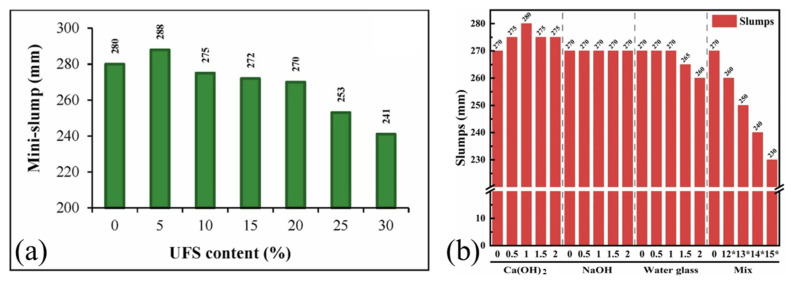
(**a**) Effect of fine-grained sediments substitution on the slump of cement paste [28]. (**b**) The slumps of filling cementitious materials (* indicates 0.5 wt% NaOH-doped) [29].

Pervious concrete using deposited fine sand or micronized powder exhibits similar zero-slump behavior [24]. Hamideh et al. [28] showed that increasing fine-grained sediments raises porosity and reduces free water, significantly decreasing slurry fluidity (Figure 13a). The type and dosage of activators also strongly affect workability [29] (Figure 13b). For example, 1 wt% Ca(OH)_2_ improves the slump, which slightly drops at 2 wt%. NaOH has a limited impact, while increased water glass sharply reduces fluidity due to coagulation and viscosity, reaching a slump as low as 230 mm with NaOH addition. These results underscore the sensitivity of sediment-based systems to activator selection and dosage.

### 4.3. Shrinkage Behavior

**Figure 14:**
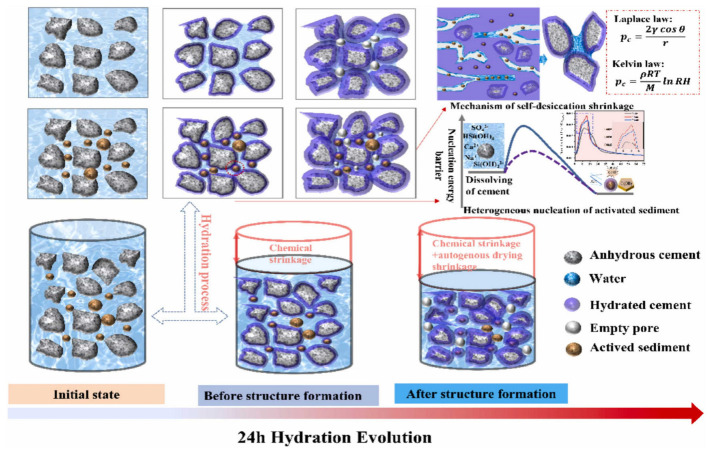
Schematic representation of the hydration process and early shrinkage changes in cement partially replaced by fine-grained sediments in the evolution of 24 h hydration [90].

**Figure 15:**
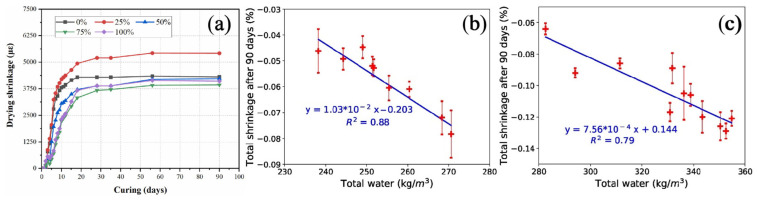
(**a**) Drying shrinkage of alkali-excited slag/fly ash mortar with different ultrafine dredged sand contents [45]. (**b**) Total shrinkage of sediment-sand concrete after 90 days versus total water in the mixture [38]. (**c**) Total shrinkage of deposited fine sand concrete after 90 days versus total water in the mixture [38].

Mechanically ground river sediments exert a dual effect of heterogeneous nucleation and microaggregate filling, which promotes hydration, refines pores, and densifies the matrix [90]. Their pozzolanic activity accelerates C–S–H formation, but reduced internal humidity and pore size lead to increased early self-shrinkage (Figure 14). In alkali-activated systems, ultrafine dredged sand affects drying shrinkage based on dosage: at 25%, 90-day shrinkage drops by 27%, but increases by up to 80% when the dosage exceeds 50% [45] (Figure 15a). Additionally, high clay content, water absorption, and low stiffness in fine sediments raise water demand and aggravate shrinkage [38,120] (Figure 15b). Thus, optimizing micronized powder dosage and precursor type is essential to control shrinkage while leveraging filling and activation benefits.

### 4.4. Fundamental Mechanical Properties

In this section, we summarize recent experimental data in Table 4 on the effects of three sediment utilization pathways—substitution of fine aggregates, substitution of cementitious materials, and synergistic modification with admixtures—on the basic mechanical properties of concrete (compressive and flexural/splitting strength), and extract the following key insights:

(1) Fine aggregate substitution: Ultrafine dredged sand can increase strength by 3–10% at a 20–50% replacement ratio; above 60%, insufficient paste coating causes a sharp strength reduction.
(2) Cementitious material substitution: Flash-calcined or alkali-activated sediments exhibit significantly enhanced pozzolanic activity, and at a 10–20% replacement of OPC or GGBS, compressive strength can increase by up to 17%, showing a “low dosage, high efficiency” effect.
(3) Synergistic additives: Sediments combined with red mud, slag, or similar materials can achieve strength gains through “activation and densification” mechanisms; some systems (e.g., YRS-GGBS-RM ground polymer) show strength improvements over 20%.

Overall, the mechanical contribution of sediments is nonlinear, and the optimal dosage depends on particle gradation, activation method, and matrix type. Research is shifting from basic feasibility studies toward multi-scale synergistic mechanisms and engineering applications, laying the foundation for standardization and durability assessments.

**Figure 16:**
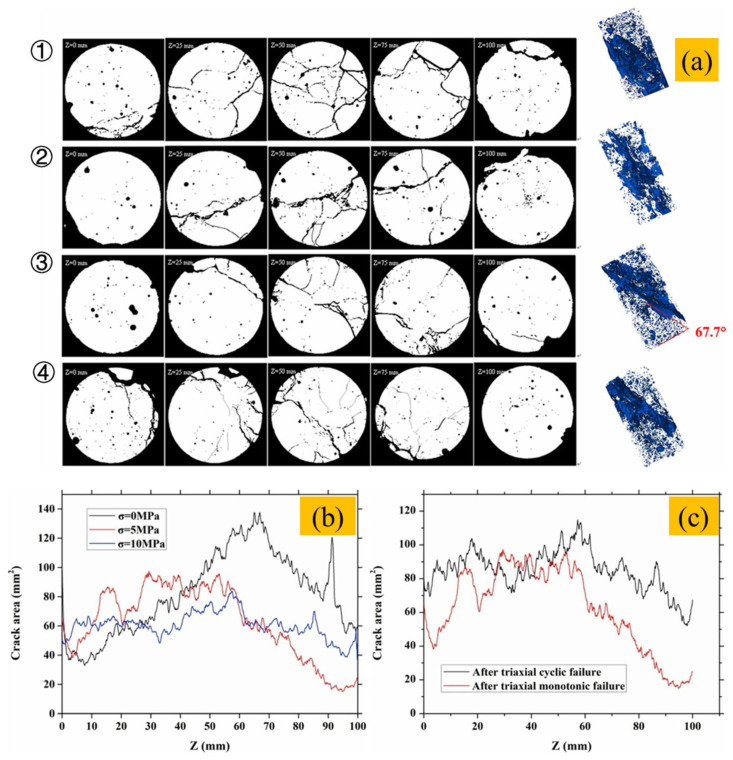
(**a**) Three-dimensional structure of CT binarized images of dredged sand concrete specimens at different heights *z* under triaxial monotonic loading (① σ_3_ = 0 MPa; ② σ_3_ = 5 MPa); ③ σ_3_ = 10 MPa; ④ Triaxial cyclic load test σ_3_ = 10 MPa); (**b**) Dredged sand concrete specimens loaded monotonically in three axes (σ = 0 MPa, 5 MPa and 10 MPa); (**c**) Evolution of cracks and pore areas along different heights z after damage by triaxial monotonic and cyclic loading of dredged sand concrete specimens (σ = 5 MPa) [119].

Engineering concrete structures (e.g., locks, dams, long-span bridges) are exposed to complex triaxial stresses and cyclic loads [121,122,123], making it critical to assess the stability of sediment-admixed systems. Luan et al. [119] used CT reconstruction to study the triaxial monotonic and cyclic loading behavior of dredged sand concrete (Figure 16a). As confining pressure decreases, fracture volume and damage increase significantly (Figure 16b); cyclic loading under the same pressure causes larger fracture zones than monotonic loading, indicating that both confining pressure and loading path influence microcrack evolution and strength degradation (Figure 16c). Additionally, inert inclusions such as shell fragments, wood chips, and slag in sediments form weak interfaces and local porosity, offsetting some strength gains [39,124,125]; however, overall porosity and carbonation depth remain comparable to control concrete [20]. Particle size also plays a key role: sandy sediments have limited impact, whereas fine sedimentary sand enhances pervious concrete strength via combined filling and activation effects [24]. In conclusion, ensuring strength and durability under complex loading requires optimized sediment activation, impurity control, and tailored particle gradation.

### 4.5. Durability Performance

Existing studies generally indicate that moderate activation or graded utilization of river sediments can enhance the durability of cementitious materials through various mechanisms.

(1) Early-stage water absorption/pore structure regulation

**Figure 17:**
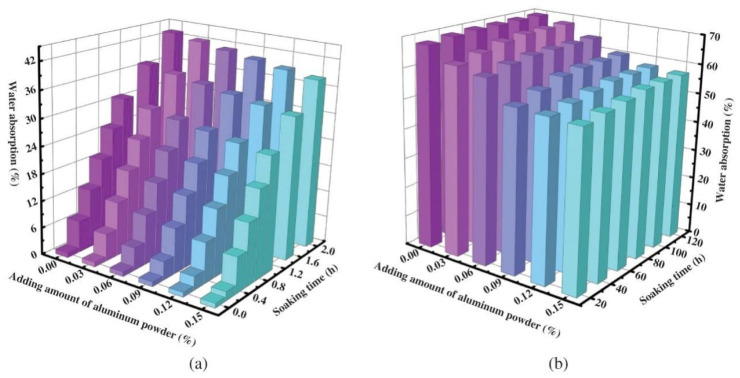
Effect of Aluminum Powder Addition on Moisture Content of AAC Blocks (**a**) 0–2 h; (**b**) 5–120 h [25].

Deposited fine sand with high specific surface area and hydrophilicity absorbs over 50% of its final water content within 0–0.2 h, followed by dominant water retention [25] (Figure 17). This “fast-absorbing-slow-releasing” behavior reduces hydration heat and optimizes pore structure, offering new potential for durability design.

(2) Sulfate attack resistance

**Figure 18:**
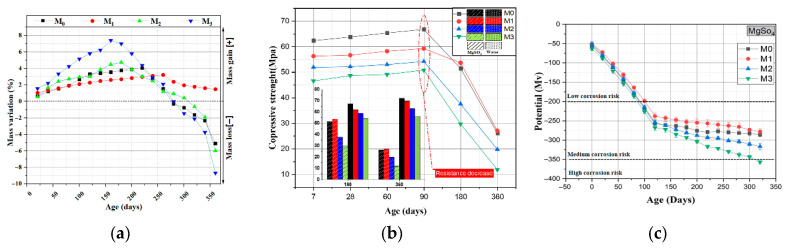
(**a**) Mass change in concrete in 5% MgSO_4_ solution; (**b**) Compressive strength of concrete in 5% MgSO_4_ solution and strength difference between mortar in MgSO_4_ and water; (**c**) Potential change in 5% MgSO_4_ solution (M0: control; M1: 15% fine-grained sediments; M2: 20%; M3: 30%) [48].

Mortar (M1) with 15% calcined fine-grained sediments replacing cement showed the lowest mass loss in 5% MgSO_4_ solution, the most stable potential, and strength gain within 90 days due to caliche/gypsum filling; degradation appeared only after 360 days, still outperforming higher substitution ratios [48] (Figure 18a–c). Calcined sediments containing 17% CaO continue to form C–S–H/C–A–H through pozzolanic reactions, enhancing matrix densification and inhibiting sulfate attack [70,126,127].

(3) Freeze–thaw durability

The compressive strength of sediment-based AAC with antifreeze remained ≥3 MPa after 15 freeze–thaw cycles [25]; concrete with 20 wt% coarse sediment substitution met XF2 requirements, with only 1.33–2.5% mass loss after 50 cycles [109].

(4) Chloride ingress resistance

High chloride permeability compromises long-term durability [128]; however, self-compacting lightweight aggregate concrete with dredged sediment and 28-day resistivity >20 kΩ·cm shows strong chloride resistance. A lower water-cement ratio further reduces cracking and mass loss [110]. Fine sand concrete from the Yellow and Liaohe Rivers also exhibited good impermeability [47].

Overall, sediment mineralogy, activation method, and particle gradation determine its durability performance. Proper calcination or mechanical activation and optimized dosage can significantly improve impermeability, erosion resistance, and freeze–thaw stability. In contrast, excessive replacement or untreated impurities may cause long-term strength loss. Future work should target durability under coupled degradation conditions to better define the limits of sediment use in high-performance concrete.

### 4.6. Hydration Kinetics and Microstructural Evolution

#### 4.6.1. Thermogravimetric Analysis

**Figure 19:**
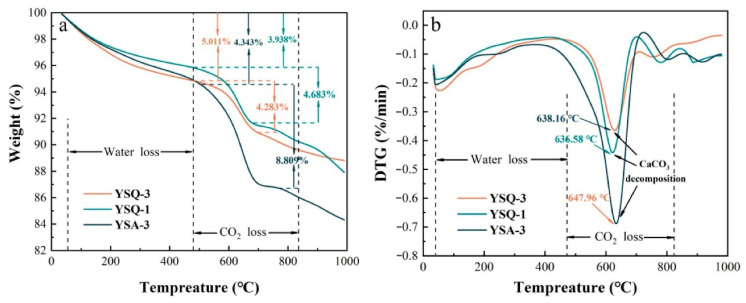
TG (**a**) and DTG (**b**) curves of 28-day YSQ–1, YSQ–3 and YSA–3 specimens. (YSQ-1: control, 100 wt% FA and 10 wt% Ca(OH)_2_; YSQ-3: 90 wt% FA, 10 wt% YRS and 10 wt% Ca(OH)_2_; YSA-3: 90 wt% FA, 10 wt% YRS and 15 wt% Ca(OH)_2_) [27].

Thermogravimetric analysis (TG) and derivative thermogravimetric analysis (DTG) are used to assess the thermal stability of sediment-replaced cementitious materials and the decomposition behavior of their components, with results shown in Figure 19. As the temperature rises, cement components gradually dehydrate and lose mass (Figure 19a). The DTG curve reflects decomposition rates, with each peak indicating a specific hydration product’s breakdown (Figure 19b). Corresponding mass loss events and their causes are summarized in Table 5.

#### 4.6.2. Microscopic Properties

(1) Scanning electron microscope

**Figure 20:**
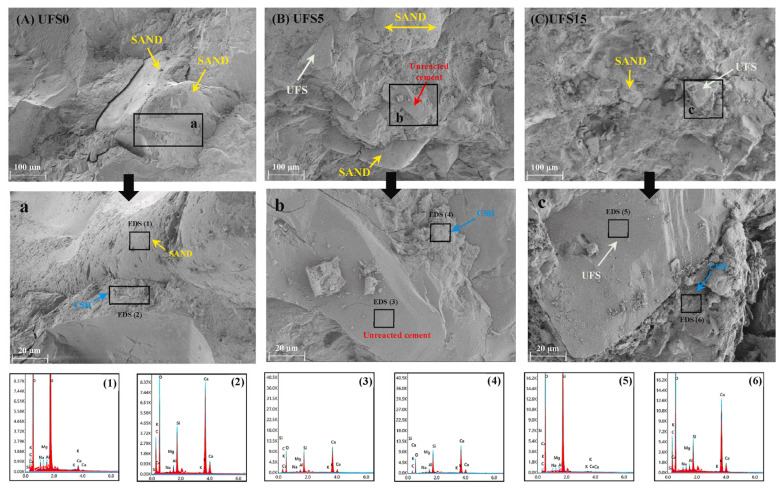
SEM-EDS images of 28-day self-compacting mortar samples: (**A**) control; (**B**) 5% deposit replacement cement; (**C**) 15% deposit replacement cement [28].

When sediment replaces natural or manufactured sand, its effect on the matrix depends on both physical filling and hydration reactivity. From a hydrochemical perspective, Hamideh et al. [28] reported that a 15% replacement ratio increased internal microcracking due to low sediment reactivity, limited hydration product formation, and weak interfacial bonding (Figure 20). This defect can damage mechanical integrity and reduce durability, especially under complex stresses. Conversely, Huang et al. [47] found that replacing 25% of manufactured sand with dredged sediment improved the interfacial transition zone by increasing concrete density, reducing porosity, and refining pore size distribution. These microstructural enhancements improve mechanical performance and durability, including resistance to chloride penetration, freeze–thaw cycles, and chemical erosion. These findings are not contradictory but reflect the dual nature of sediment substitution: it is influenced by both physical effects (filling and grading) and chemical limitations (reactivity and bonding), which are closely tied to the sediment’s source and mineralogy. Therefore, sediment replacement assessment should include pore structure and hydration chemistry analyses, correlated with macro-scale mechanical and durability indicators.

**Figure 21:**
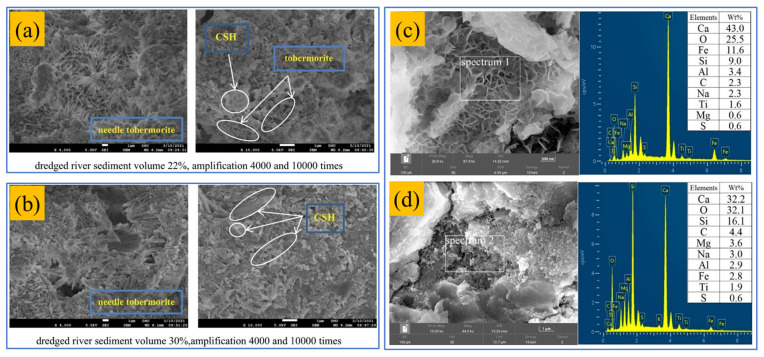
(**a**) Mortar with 22% dredged river mud content [25]; (**b**) mortar with 30% dredged river mud content [25]; and (**c**,**d**) dredging mud content is 40% and 60% of red mud geopolymer cement base material: natural curing (**c**) [111] and (**d**) the standard maintenance [111].

To evaluate the feasibility of partially replacing cementitious materials with sediment, it is essential to examine changes in hydration product morphology and chemistry. At low dosages, slender needle-like hydrates form (Figure 21a); at higher dosages, coarser needles form a denser skeleton (Figure 21b) [135], enhancing load-bearing capacity and fracture resistance. These coarser crystals enhance erosion resistance and can maintain or improve compressive strength [25]. EDS surface scans further show that high Ca content in raw sediment promotes extensive C–A–S–H gel formation, indicating that elemental migration and curing conditions reshape the gel structure (Figure 21c) [111]. The resulting gels improve both early and long-term strength and enhance resistance to ionic diffusion and carbonation. Standard curing activates low-reactivity components, forming Si, O-rich (N,C)–A–S–H gels that densify the matrix (Figure 21d), reduce permeability, and improve durability in harsh environments. In summary, the advantages of using sediment to replace cementitious materials arise from both morphological densification and chemical activation, and their synergy governs the enhancement of mechanical and durability performance. Optimized substitution ratios refine microstructure and produce more durable, mechanically robust concrete.

(2) Pore structure characterization

**Figure 22:**
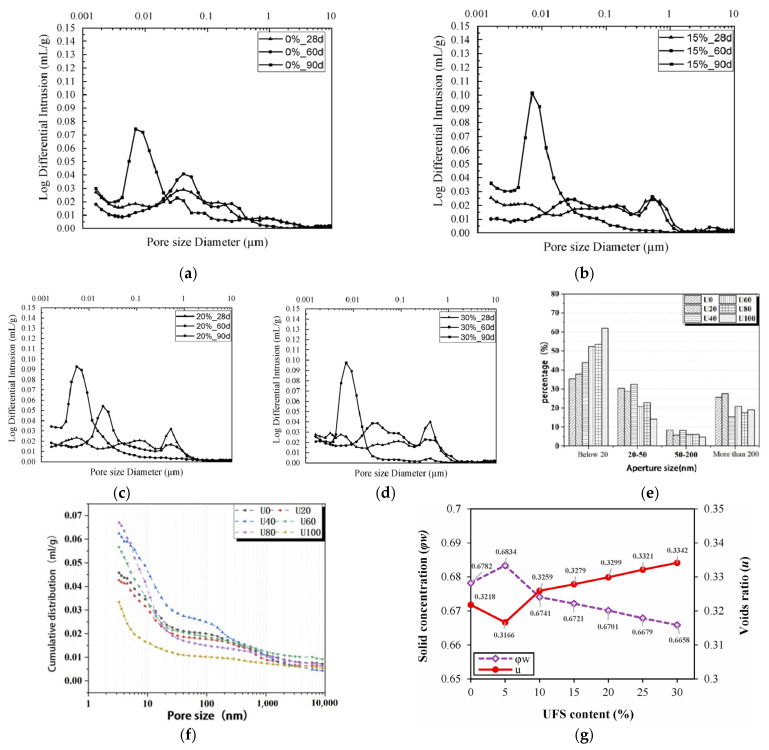
Porosity of mortar at different maintenance ages and at different sediment substitution rates: (**a**–**d**) substitution of cementitious materials [48]; (**e**,**f**) substitution of fine sand [26]; (**g**) the effect of UFS content on the packing density (*φ_w_*) and voids ratio (*u*). UFS: ultra-fine sediment [28]. The impact of activated sediment replacing cement manifests in several aspects. Initially, alite hydration increases, but the difference in reaction degree narrows after 3 days. The CH content decreases due to the pozzolanic reaction triggered by activation [26]. Activated alumina in the sediment reacts with Ca(OH)_2_ and CaSO_4_ to form additional ettringite in early hydration [138]. During later stages, the content of mono- and hemicarbonate AFm phases increases, and Al released from activated sediments slightly raises AFm levels [78].

Ali Benkabouche [48] systematically analyzed the pore evolution of mortars with 0–30% sediment replacing cement using mercury intrusion porosimetry (Figure 22a–d). While sediments as mineral admixtures can enhance matrix density and mechanical strength [136,137], their effect on pore size distribution is relatively limited. Even moderate pore refinement improves mechanical consistency and reduces permeability, enhancing durability against freeze–thaw and chemical attack. In contrast, Jun [26] found that using Yellow River sedimentary sand to replace fine aggregate in UHPC significantly reduces the proportion of harmful and multi-harmful pores, transforming the pore structure toward less harmful types. This refinement mitigates strength loss and enhances fracture resistance, improving reliability and extending service life under harsh conditions (Figure 22e,f). However, when sediments are used as micronized powder, they compete with cement for filling space, and their lower filling capacity leads to increased porosity (Figure 22g), as confirmed by Hamideh et al. [28]. Increased porosity reduces compressive and flexural strength and durability by allowing fluid ingress and accelerating corrosion and degradation. Overall, the effect of sediments on pore structure depends on particle size, shape, and replacement mode. Achieving a balance between pore optimization and performance improvement requires fine-grading or synergistic blending strategies.

## 5. Taxonomy and Application Scenarios of Sediment-Tailored Multifunctional Concrete

According to sediment characteristics and engineering demands, researchers have developed sediment-based concrete for diverse applications, including pervious concrete, lightweight concrete, foam concrete, self-compacting mortar, polymer mortar, autoclaved aerated concrete (AAC), ultra-high-performance concrete (UHPC), and engineered cementitious composites (ECC), as shown in Figure 22. These materials function either by replacing sand or cementitious components with sediment.

### 5.1. Deposited Fine Sand as Partial Sand Replacement

(1) Pervious concrete: Compared to conventional concrete, pervious concrete has lower thermal conductivity and heat capacity, helping to mitigate urban heat island effects [24]. Beddaa et al. [39] found that finer dredged sediments, used to fully replace sand, produced denser concrete with reduced porosity and permeability and improved compressive strength.
(2) Lightweight and thermal-insulating concrete: A porous ceramic masonry mortar using Yellow River sediment and coal dust fully replaced standard sand and offered lightweight properties [23]. Zhang et al. [113] further improved the performance by incorporating biochar, enhancing water retention, water resistance, and insulation, making it suitable for green building applications.
(3) Polymer-based mortar: Maherzi et al. [101] developed a polymer mortar by replacing traditional sand with sediment. The resulting material showed enhanced chemical resistance and thermal stability, with superior durability under corrosive and thermal conditions.
(4) Autoclaved aerated concrete (AAC) and non-AAC blocks: AAC blocks made with 30–34% dredged silt, 24% cement, 10% quicklime, 30% fly ash, 2% gypsum, 0.09% aluminum powder, and a 0.5 water-to-material ratio, cured under 2.2 MPa for 6 h, achieved 4.5 MPa strength and 716.56 kg/m^3^ dry density [25]. Non-AAC blocks using 15% sediment, 48% cement, 20% lime, and the same water ratio reached 3.1 MPa and 924.19 kg/m^3^. Both met national standards.
(5) High-performance structural concrete: Ultrafine Yellow River sand enabled high-strength UHPC with a refined pore structure even at high substitution levels [26]. Yuan et al. [139] applied it to ECC, achieving full quartz sand replacement. At 75% substitution, the material showed optimal ductility, crack control, and mechanical performance, supported by finite element modeling.

### 5.2. Fine-Grained Sediment as Binder Replacement

(1) Foam concrete for sound insulation and air purification: Foam concrete combines sound insulation, low thermal conductivity, and photocatalytic performance. However, sediment addition lowers pH, weakening the foaming effect. At 40% cement replacement, the mixture achieves optimal performance: best sound insulation (1767 m/s, 9% foaming agent), lowest thermal conductivity (0.2831 W/m·K, 6% foaming agent), and excellent photocatalytic efficiency (14.1%, Bi_4_Ti_3_O_12_). Under optimized sediment and foaming agent ratios, it shows strong multifunctionality, making it suitable for green building applications [5]. Heavy metals in the sediment are well encapsulated, with low leaching even in acidic conditions. Silica fume can also be added as a pore-refining agent [38]. Jiang et al. [27] used Yellow River silt in alkali-activated fly ash foam concrete and studied the effects of sediment and alkali content on fluidity, mechanical properties, pore structure, and thermal performance, confirming its feasibility as a supplementary material.
(2) Self-compacting and high-flow mortar: Mehdizadeh et al. [28] prepared self-compacting mortar by replacing 5–15% of cement with ultrafine Yangtze River sediment. The resulting mortar maintained good flowability and mechanical strength, meeting construction needs for complex structures.
(3) Alkali-activated cementitious materials (AAMs) concrete: Li et al. [45,106] used sediment from the lower Yangtze River to prepare AAMs concrete and found that a 25% replacement rate improved strength, microstructure, and shrinkage resistance, offering good durability for green infrastructure. Yu et al. [29] combined Yellow River silt with fly ash and cement to develop alkali-activated materials for coal mine backfilling. They studied the effects of Ca(OH)2, NaOH, and water glass, revealing that the formation of C–(A)–S–H gel enhanced hydration, pore structure, strength, and workability, achieving both resource utilization and environmental benefits.
(4) Application of Sediments in Ternary Cementitious Systems: Literature confirms sediments are a promising component in ternary systems. Some studies show sediments as key ternary blend components, enhancing mechanical properties and refining microstructure. First, explorations have focused on ternary systems with calcined sediment, limestone filler, and cement. Bellara, Zeraoui, Hadj Sadok, and others [13,14,140,141] prepared low-carbon binders by combining GGBS and cement with clay dam, flash-burned, or canal sediments. By optimizing the sediment/GGBS/cement ratio and moisture content, they enhanced fluidity and strength, developing an eco-friendly hydraulic road binder (HRB). The hydration mechanism was clarified: flash-burned sediment provides activity, GGBS adds strength, and cement supplies alkalinity synergy. Furlan et al. [142] studied fly ash reinforcement in lime–cement stabilized dredged sediment. They detailed the pozzolanic and micro-filling effects of fly ash on pore structure and products, linking them to improved strength and water resistance. Second, other ternary systems combined sediments with different mineral admixtures. Zhang et al. [143] showed that mixtures of sediment, slag, and fly ash achieved good compactness and mechanical performance. Yu [29] and Wang [75] focused on Yellow River sediment, formulating alkali-activated or cementitious ternary systems (sediment–fly ash–cement) for backfill and mortar. These studies achieved high strength and workability while controlling efflorescence by lowering alkaline ion concentrations. Collectively, these works confirm the strong potential and practicality of using sediments in ternary cementitious systems.

By replacing sand or cement, sediment-based concrete enables material recycling and functional enhancement. It meets structural safety requirements and offers energy-saving, environmental, and multifunctional advantages. Current applications include urban infrastructure, green buildings, functional materials, and ecological restoration. Future work should focus on standardizing classification, dosage control, and engineering adaptability to promote broader application.

## 6. Exploring the Classification and Application Scenarios of Sediment-Based Multifunctional

Urbanization has increased domestic and industrial wastewater discharge into rivers, leading to heavy metal accumulation in dredged sediments. For sediment concrete to be safely applied, the heavy metal content in leachate must not exceed that of control concrete or inert waste limits [39].

Environmental studies show that appropriate sediment incorporation can effectively immobilize heavy metals, ensuring environmental safety. Kai [25] used inductively coupled plasma mass spectrometry to test heavy metal levels in aerated concrete blocks. Except for Cr (average 4.23 µg/L), other metals were below detection limits. Leachate tests at 12 h and 18 h showed variation within 0.1%, confirming stable immobilization and environmental safety during use.

Mostafaei et al. [144] showed that replacing part of cement and sand with zeolite achieved 85 MPa compressive strength and reduced the carbon footprint to 659.72 kg CO_2_/m^3^, supporting the feasibility of sustainable materials such as sediment. Economic analysis showed that sediment-based C30 concrete costs 41% less than conventional concrete. If the quarry is over 30 km away from the sediment site, sediment concrete can reduce the carbon footprint equivalent to 5% added cement [39]. For high-performance cementitious materials, Jun Yan [26] showed that replacing quartz sand with Yellow River ultrafine sediment in UHPC reduced primary energy use by up to 7% while maintaining mechanical strength, as assessed by EN ISO 14040 [145]. Costs were cut to 55% under full substitution, highlighting the potential of sediments in green and high-value materials.

Based on a case study in Zhengzhou, China, where Yellow River sediment replaced 25% of cement in mortar production, a life-cycle cost and carbon emission analysis was carried out. The functional unit was defined as 1 m^3^ of ready-mixed mortar, in line with industry standards, to ensure comparability of environmental and economic impacts [146]. The system boundary followed a cradle-to-gate approach, with analysis focusing on key cost and carbon emission factors [146,147,148]. Key assumptions were as follows: cement transportation distance was set at 30 km, with a cost of 0.6 RMB/kg; Yellow River sediment transportation distance was set at 20 km, classified as waste utilization with no raw material cost. The results indicated that using Yellow River sediment reduced cost by about 24.2% and carbon emissions by 24.8% per cubic meter of mortar, highlighting its considerable economic and environmental potential.

In summary, sediment used in cementitious materials can significantly reduce production costs while ensuring environmental safety. It also offers clear advantages in carbon emission reduction and ecological performance, supporting the green transformation of construction materials and high-value utilization of solid waste. Future work should focus on assessing long-term environmental stability under complex conditions to facilitate engineering applications and industrialization of sediment-based materials.

## 7. Conclusions and Outlook

### 7.1. Conclusions

Based on an extensive review of the literature and experimental results, this paper systematically evaluates the use of river sediments in concrete. Key findings and research directions are as follows:

(1) The resource properties and application potential of river sediments were evaluated. With wide particle size distribution, high SiO_2_ content, and cementitious potential, sediments can replace natural sand, gravel, and partial cement as fine aggregates, lightweight aggregates, or pozzolanic materials after proper grading and decontamination.
(2) The activation mechanisms and property enhancement strategies were summarized. Synergistic activation with alkali (NaOH or Ca(OH)_2_) and water glass promotes full consumption of Ca(OH)_2_ and formation of dense C–S–H/C–A–S–H networks. Thermal treatment above 650 °C enhances the activity of clay minerals like kaolinite and chlorite, though activity declines beyond 900 °C. Mechanical milling and incorporation of additives such as biochar and fibers form a “micro-cement + filler” composite structure, improving 28-day compressive strength by 10–20%.
(3) Optimized mix design strategies were established. Combining minimum paste theory with packing density optimization reduces paste volume by 8–12% while maintaining a slump ≥ 180 mm. Optimal mechanical and durability performance is achieved when the sediment replacement rate is controlled at 25% ± 5%.
(4) The environmental and economic benefits of sediment reuse were confirmed. Life cycle assessment shows that replacing 30% of cement with sediment can reduce CO_2_ emissions by approximately 180 kg/m^3^ and material costs by 10–15%, offering substantial ecological and economic advantages.

### 7.2. Outlook

(1) A unified evaluation system for sediment grading, activation level, and application classification is urgently needed to support large-scale engineering deployment.
(2) Multi-scale coupling mechanisms should be explored using in situ neutron/X-ray techniques and molecular dynamics to uncover sediment–cement interface reactions and long-term degradation behavior.
(3) Future work should study the durability of mud–sand concrete under coupled degradation, focusing on synergistic effects between carbonation and other stresses such as chloride intrusion, dry–wet cycling, or mechanical loading.
(4) Low-energy activation processes such as microwave–alkali synergy should be developed to reduce energy use and secondary emissions.
(5) Future research should focus on intelligent functionalization, including sediment-based materials with temperature control, phase-change energy storage, and photocatalytic functions to achieve multifunctionality and high-value applications.

## Figures and Tables

**Table 1:** Moisture content, water absorption, and density of river sediments.

		Water Content (%)	Absorption (%)	Density (g/cm^3^)
Conventional aggregates [39]	Sand	0.081	4.7	2.41
	Gravel	0.065	2.3	2.53
Neuilly raw sediment [39]	Sand	N.D.	3.0	2.45
	Gravel	10.69	1.8	2.53
Puteaux raw sediment [39]	Sand	N.D.	3.6	2.46
	Gravel	6.43	2.6	2.47
Preparation of lightweight aggregates [22]	Gravel	N.D.	5.5–9.5	1.01–1.38
Preparation of ceramic granules [23]	Gravel	N.D.	5.9	0.879
Seine Basin [24]	Sand	N.D.	N.D.	2.24–2.41
	Gravel	N.D.	3.7–17.2	2.05–2.41
Yangtze River ultra-fine dredged sand [45]	Sand	N.D.	4.5	2.69

Note: N.D. (Not determined).

**Table 2:** Chemical composition of river sediment and cement.

Sports Event	SiO_2_ (%)	Al_2_O_3_ (%)	CaO (%)	Fe_2_O_3_ (%)	Na_2_O (%)	K_2_O (%)	MgO	LOI
Cement 1 [29]	21.49	5.24	64.16	2.89	0.76	0.42	2.12	1.0
Cement 2 [5]	18.093	5.882	64.394	3.904	N.D.	1.219	1.944	N.D.
Yellow River sediment 1 [29]	68.73	11.08	7.56	3.64	1.76	3.21	2.11	2.85
Yellow River sediment 2 [23]	60.5	15.9	9.96	4.77	1.56	2.81	3.18	N.D.
Yangtze River sediment 1 [47]	68.2	11.4	5.66	3.22	1.88	2.31	2.46	N.D.
Yangtze River sediment 2 [45]	68.74	11.4	5.56	3.20	1.84	2.3	2.42	N.D.
Mud and sand sediment 1 [46]	47.50	15.60	10.20	6.70	0.30	1.90	2.40	15.1
Mud and sand sediment 2 [47]	75.717	11.988	1.59	4.971	0.029	1.857	0.951	N.D.
Mud and sand sediment 3 [48]	33.25	8.65	13.1	3.89	0.45	149	0.75	N.D.
Mud and sand sediment 3 calcined at 750 °C [48]	43.45	10.30	15.4	4.95	0.63	1.84	0.91	N.D.
Mud and sand sediment 4 [25]	56.93	18.98	2.14	12.05	0.70	4.07	2.07	4.13
Fly ash 1 [18]	50.6	27.2	2.8	7.0	0.5	2.6	0.97	1.14
Fly ash 2 [25]	53.7	27.3	7.7	5.70	0.7	0.12	1.8	0.40

Note: LOI is short for loss on ignition at 1000 °C; N.D. = not determined.

**Table 3:** The design method of the concrete mix proportion.

Method	Name	Principle	Features
Aggregate-based method	Compressive Packing Model (CPM) [95]	This approach uses virtual packing density to model particle combinations and improve packing efficiency.	It requires full particle size distribution data.
	Andreasen and Andersen model [26,96,97]	This method determines component ratios by optimizing a continuous particle size distribution curve.	It provides more precise control than the traditional residual fitting method.
	Absolute Volume Method [98,99,100]	This theory derives component ratios from aggregate volume.	It is complex and requires separate volume data for each material.
	Packing Density Modeling (PDM) [101]	This model predicts mixture density using energy input, material densities, and particle sizes.	It avoids the separate calculation of packing parameters and achieves relatively high accuracy.
Slurry-based method	The minimum paste theory [102].	This theory considers concrete as an aggregate skeleton coated with the minimum paste.	Reducing paste volume improves volumetric stability and crack resistance while maintaining workability and strength.

**Table 4:** Comprehensive comparison of the mechanical properties of concrete/mortar by river sediment under different replacement strategies.

	Setup and Level	Sediment Sources/Treatment	Range of Replacement Rates (%)	Optimal Substitution Rate (%)	Change in Compressive Strength	Change in Bending/Splitting Strength	Remarks/Key Conditions
**Sediment as Fine Aggregate Replacement Systems**							
[28]	Self-compacting mortar	Ultrafine sediments (unactivated) in the Yangtze River Estuary	5–30	5	45.88 → 43.51 MPa ↓ 5%	8.02 → 8.84 MPa ↑ 10%	Rapid decrease in strength after >15%
[45]	Alkali-inspired system mortar	Ultrafine sediments in the Yangtze River + Ground Granulated Blastfurnace Slag: Fly Ash = 3:7	25–100	25	25.67 → 26.36 MPa ↑ 3%	4.50 → 5.08 MPa ↑ 13%	Activation system, >50% significant decrease
[45]	Alkali-inspired system mortar	Ultrafine sediments in the Yangtze River + Ground Granulated Blastfurnace Slag: Fly Ash = 7:3	0–100	25	59.18 → 61.2 4 MPa ↑ 3%	9.09 → 9.34 MPa ↑ 3%	—
[26]	Ultra high-performance concrete	Ultrafine sediments of the Yellow River + Fine sand	0–100	80	100.3 → 104.3 MPa ↑ 4%	15.5 → 16.9 MPa ↑ 9%	Optimum 80% sand mixing ratio
[106]	Alkali-inspired concrete	Ultrafine sediments in the Yangtze River	0–100	50	49.89 → 54.60 MPa ↑ 9%	—	—
[39]	Ordinary concrete	Seine River sediments (completely replacing coarse and fine aggregates)	100	—	Requires ↑ 5% cement to reach C30 grade strength	—	Technical feasibility, economic assessment
[47]	Ordinary concrete	Yangtze River dredged sand and machine-made sand concrete	0–50	25	41.48 → 65.50 MPa ↑ 57.9%	—	—
**Sediment–Cement Binder Systems**							
[107]	OPC mortar	(Flash calcination) calcined dredging sediments	20–40	20	64.74 → 71.46 MPa ↑ 10%	—	Flash calcination activation is significant
[27]	Foam Concrete	Yellow River sludge (Ca(OH)_2_ activation)	5–20	5	4.32 → 3.82 MPa ↓ 11%	—	High substitution rate intensity falls
[112]	Ground polymer mortar	Yellow River Silt Replacement Ground Granulated Blastfurnace Slag + Red mud	10–40	Standard maintenance: 10; High temperature water bath maintenance: 20	68.10 → 80.00 MPa ↑ 17% (Standard maintenance); 22.06 → 65.43 MPa ↑ 17.47 (High temperature water bath maintenance)	—	Standard maintenance; High temperature water bath maintenance, same trend
[112]	Porous ground-polymerized concrete	Yellow River Silt Replacement Ground Granulated Blastfurnace Slag + Red mud	10–40	10	24.35 → 26.25 MPa ↑ 8%	—	—
[111]	Alkali-inspired system mortar	Yellow River sediment + Red mud	20–80	40	16.80 → 26.52 MPa ↑ 57.86 (Natural Curing); 39.87 → 48.67 MPa ↑ 22.07% (Standard Curing)	—	The compressive strength of 28 d under standard curing can be increased by 82~132% to that under natural curing

**Table 5:** Analysis of causes of mass loss at different temperatures.

Temperature Range(°C)	Main Causes of Quality Loss	Note
50–100	Free water evaporation	Corresponds to the weight loss peak at ~100 °C in the DTG curve, initial physical weight loss, which is present in all samples [129].
100–200	C–S–H and C–A–S–H initial dewatering; AFt, AFm dewatering	The appearance of a distinct weight loss peak indicates the formation of reaction products that enhance structural strength [130,131].
200–500	C–S–H and C–A–S–H continuous dewatering	The process continues and affects the mechanical strength of the samples. From this stage, it is possible to determine the promotion or inhibition of gel generation by the addition of river sediments [27].
400–500	Ca(OH)_2_ dehydroxylation reaction (chemistry)	Ca(OH)_2_ is involved in the reaction or consumed by carbonation [29]. It is possible to determine whether river sediments promote CH production based on the amount of CH consumed at this stage of the process. Furthermore, this densification inhibits penetration of aggressive agents, directly enhancing concrete durability.
~630	CaCO_3_ decomposition	Related to the carbonation reaction, increased carbonation products enhance strength and densification [132,133,134].
500–850	Decomposition of heat-stabilized components such as calcium carbonate	The degree of overall mass loss indicates the extent to which sediment affects cement hydration.

## Data Availability

The authors confirm that the data supporting the findings of this study are available within the article.
